# Procedures of recruiting, obtaining informed consent, and compensating research participants in Qatar: findings from a qualitative investigation

**DOI:** 10.1186/1472-6939-15-9

**Published:** 2014-02-04

**Authors:** Amal Killawi, Amal Khidir, Maha Elnashar, Huda Abdelrahim, Maya Hammoud, Heather Elliott, Michelle Thurston, Humna Asad, Abdul Latif Al-Khal, Michael D Fetters

**Affiliations:** 1 Department of Family Medicine, University of Michigan, Ann Arbor, MI 48104, USA; 2 Weill Cornell Medical College in Qatar, Education City, PO Box 24144, Doha, Qatar; 3 Department of Obstetrics and Gynecology, University of Michigan, 1500 E. Medical Center Drive, L4000 Women’s, Ann Arbor, MI 48109, USA; 4 Department of Health Management and Policy, School of Public Health, University of Michigan, Ann Arbor, MI 48109, USA; 5 Department of Medical Education, Hamad General Hospital, PO Box 3050, Doha, Qatar

**Keywords:** Research ethics, Recruitment, Informed consent, Cultural competence, Middle East, Research participation, Vulnerability, Confidentiality, Qualitative research, Research compensation

## Abstract

**Background:**

Very few researchers have reported on procedures of recruiting, obtaining informed consent, and compensating participants in health research in the Arabian Gulf Region. Empirical research can inform the debate about whether to adjust these procedures for culturally diverse settings. Our objective was to delineate procedures related to recruiting, obtaining informed consent, and compensating health research participants in the extremely high-density multicultural setting of Qatar.

**Methods:**

During a multistage mixed methods project, field observations and qualitative interviews were conducted in a general medicine clinic of a major medical center in Qatar. Participants were chosen based on gender, age, literacy, and preferred language, i.e., Arabic, English, Hindi and Urdu. Qualitative analysis identified themes about recruitment, informed consent, compensation, and other research procedures.

**Results:**

A total of 153 individuals were approached and 84 enrolled; the latter showed a diverse age range (18 to 75 years); varied language representation: Arabic (n = 24), English (n = 20), Hindi (n = 20), and Urdu (n = 20); and balanced gender distribution: women (n = 43) and men (n = 41). Primary reasons for 30 declinations included concern about interview length and recording. The study achieved a 74% participation rate. Qualitative analytics revealed key themes about hesitation to participate, decisions about participation with family members as well as discussions with them as “incidental research participants”, the informed consent process, privacy and gender rules of the interview environment, reactions to member checking and compensation, and motivation for participating. Vulnerability emerged as a recurring issue throughout the process among a minority of participants.

**Conclusions:**

This study from Qatar is the first to provide empirical data on recruitment, informed consent, compensation and other research procedures in a general adult population in the Middle East and Arabian Gulf. This investigation illustrates how potential research participants perceive research participation. Fundamentally, Western ethical research principles were applicable, but required flexibility and culturally informed adaptations.

## Background

With globalization, the growing numbers of international partnerships in health services research pose challenges and opportunities [1]. For international investigations, Western researchers such as the authors are accountable in their home institutions to Western ethical principles. When there are different standards between Western institutions and host country collaborators, tension can arise over preferred approaches [2]. For example, when U.S. researchers participate in international projects, they are bound to U.S. regulations [3], and the required approach to informed consent becomes based on Western ethical standards [3,4]. However, there are concerns about the appropriateness of applying a ‘Western’ approach to recruitment and consent procedures among culturally diverse groups in non-Western countries [3-6] and by extension among culturally diverse groups within Western societies [7]. Areas of debate address: pressure to participate [3,8], limited comprehension of informed consent [3,5,8], verbal versus written documentation of informed consent [3,5,8], language and literacy barriers [3,8], confidentiality [9], and individual vs. community decision-making processes [3,5,8].

While some researchers advocate for universal ethical principles in research [5,10-13], others argue that standards must be adjusted for culturally diverse settings [4,7,8]. In support of the latter, Fadare and Porteri illustrate how Nigeria incorporates cultural considerations for human subjects in its national research guidelines in accordance with the Helsinki Declaration of the World Medical Association and the International Ethical Guidelines for Biomedical Research of the Council for International Organizations of Medical Sciences [2]. In addition, empirical research illustrates that researchers on the ground feel cultural influences need consideration. For example, in a study with American researchers conducting research in developing countries, Dawson and Kass [3] found that most respondents believed U.S. regulations should allow more flexibility in informed consent regulations compared to prevailing Western standards. In a survey of 203 researchers conducting human subject research in developing countries, Hyder and Wali [5], found that written consent was not used by approximately 40% of the researchers. Solving these challenges requires culturally informed strategies to obtain and document informed consent [3,8].

In the Middle East and Arabian Gulf, only a very limited literature informs the debate about culturally adapted procedures for recruiting, obtaining informed consent, and compensating health research participants. A search of PubMed’s extensive database of medical literature, of Google and Google Scholar’s archives, Global Health Database, Articles Plus, PsycInfo, Social Sciences Citation Index/Web of Science, and Scopus’s depository of resources revealed only 11 articles addressing methodological procedures of recruitment and informed consent for research in this region. Emanating from Qatar, Oman, Egypt, and other countries in the Middle East and Arabian Gulf, these papers provide insight on seven topics: (1) influence of cultural idiosyncrasies on the level of disclosure and the informed consent process in clinical care [14]; (2) differences in interpreting ethical research principles [15-17]; (3) challenges implementing Western research principles, particularly informed consent [18,19]; (4) difficulty of obtaining informed consent from illiterate individuals [20]; (5) difficulty with recruitment [9,21], (6) perceptions about informed consent specific to biobanking research [22], and (7) the importance of utilizing the expertise of community and non-scientific leaders to ensure recruitment and consent procedures are consistent with the host country’s cultural, political, and social practices [23]. None of the articles address participants’ reactions to being offered compensation for participating in research.

Surprisingly, only two of these 11 papers provide a glimpse of outcomes of recruitment and informed consent procedures for research with Arab populations. Kahan and Al-Tamimi (2009) present data about their research recruitment experience with Arab Americans in San Diego, California with adults aged 18–29 using snowball sampling, flyers, presentations to university campus organizations, and through graduate research assistants; nearly two-thirds of participants had some college or graduate education. To our knowledge, the only previous study reporting empirically on experience with obtaining informed consent for research in the Middle East was conducted by Nakkash and others (2009) with Palestinian refugees 10–14 years of age in Lebanon. Based on their field observations, the authors identify challenges related to disclosure, comprehension, capacity, voluntariness and consent, and argue for cultural adaptations while upholding the intended ethical principles [18].

The purpose of this paper is to address the gap in the literature on procedures for recruiting, obtaining informed consent, and compensating participants in health research in the Middle East and Arabian Gulf Region. The findings are based on the research team’s field observations and interviews of participants during one stage of a multistage research project about health care quality assessment in Qatar.

## Methods

### Design

This research utilized an ethnographic approach to conduct a qualitative study as one component of a multistage, mixed methods parent study focused on developing a self-administered healthcare quality assessment instrument in the four languages of Arabic, English, Hindi, and Urdu [1]. Briefly, the first stage of the parent study involved cultural adaptation of the adult survey of the Consumer Assessment of Healthcare Providers and Systems Survey (CAHPS) [24]. The second stage of the parent study involved qualitative interviews regarding patient perspectives on health care quality. Based on our field observations during the second stage, extensive field notes were collected about procedures of recruiting, obtaining informed consent and compensating participants of the study. The field notes and comments about recruitment, informed consent, compensation and other field procedures from the interviews served as the two sources of data for this project. Human subjects approval for the study was granted from the University of Michigan, Weill Cornell Medical College in Qatar, and Hamad Medical Corporation (HMC) [1,24].

### Setting

This study was conducted in the extremely high-density multicultural setting of Doha, Qatar [25]. Qatar has a population of approximately 1.9 million people [26] with the majority comprised of expatriate workers from all over the world temporarily residing in Qatar [20,26,27]. Health care services at HMC are subsidized and considered a governmental public service for Qatari nationals and Qatari residents. HMC, the premier health care provider in Qatar, served as the specific data collection site.

### Data collection instruments

The project instruments included a recruitment script, an interview guide, and a single sheet containing both information about the research and a waiver of written informed consent. All documents were developed with the goal of a fifth grade reading level. All documents (except the waiver of informed consent template that was already available in Arabic and English) were developed in English and then independently translated. Each translated document was reviewed by two translators who compared the translations to develop a best final version.

### Sampling and enrollment procedures

We targeted the recruitment of approximately 80 subjects, about 20 participants per language for in-depth interviews. Our study employed all-female research assistants (RAs) because it is culturally more acceptable in public places in Qatar, including healthcare settings, for women to move into both male and female waiting areas, and thus more efficient for the study. The RAs wearing an official white research coat approached patients or family members in the outpatient waiting room of the clinic to inform them about the study. Inclusion and exclusion criteria are depicted in Table 1. Recruitment sought roughly equal representation within each group by literacy (low versus high). After careful consideration of primary populations in the region [1], low literacy was defined as 9^th^ grade and below and high literacy as some high school education or greater. While most linguistic groups corresponded with specific cultural groups, English serves as the lingua franca for many cultural groups in Qatar that speak English as a second language. The team exercised maximum variation sampling [28] to achieve cultural diversity in the English language group to avoid over-representation of any specific groups. Eligible individuals who enrolled for interviews gave verbal consent that was audio recorded. These individuals received the information/waiver of written informed consent sheet at this time. This sheet included a statement that participation in the study would not impact participants’ receipt of health care. Additionally, the RAs emphasized this point when recruiting and interviewing participants. Interviews ranged from 15–60 minutes in duration.

**Table 1:** Inclusion and exclusion criteria

**Inclusion criteria**	**Exclusion criteria**
• Speaks as first language the target language—primary language was defined as a language that the participant grew up speaking and/or reading from his or her childhood, or as determined by sociocultural norms such as work environment of his or her home country	• Declines interest in participating
	• Has a severe debilitating illness precluding participation
	• Does not speak the target language
• Has lived in Qatar for at least 12 months in the past 3 years	
• Provides verbal informed consent	

### Data collection procedures

#### Field observations

Field observations served as the primary source of data for this study and were conducted under the human subjects approvals for the project. The RAs collected field jottings [29] about the events, behaviours, and environment during recruitment and data collection procedures that they expanded into full field notes after return to the research office. They recorded the number of individuals who were approached, declined, enrolled, excluded, and reasons for exclusions. The RAs discreetly recorded gender, cultural affiliation, approximate age, and reason for declining that individuals volunteered without prompting. No identifiable personal health information was collected from individuals who declined. For individuals who were enrolled, the RAs tracked how many of them retained the information/waiver of written informed consent sheet, how many provided contact information, and how many spontaneously shared their name with the RA as an indication of trust.

#### In-depth interviews

The interviews were conducted primarily for the parent study on developing a culturally adapted survey on health care quality. The interview guide included questions about the individual’s health and illness experiences, healthcare practices, challenges encountered to receive health care, recommendations for improvement in services, and demographic information. While the interviews were not focused on procedures of recruiting, obtaining informed consent, and compensation, many interviews provided supplementary information to the field observations. The RAs interviewed participants in the outpatient waiting area of the general medicine clinic of HMC. The waiting area was chosen as the location for interviews for two reasons after a careful discussion amongst team members. First, cultural norms in Qatar dictate that interactions between genders occur in public space in view of others except for purely medical reasons or necessity depending on the task. The mostly Muslim and all-female RAs felt it would be culturally inappropriate for them to be in a private room with a man. Second, participants were interviewed while waiting for their doctor’s appointments. Since the hospital uses a number system, participants preferred to stay in the waiting area with full visibility of the number display used to call patients. When available and deemed to be culturally acceptable, the RAs occasionally used vacant patient rooms for interviews.

### Compensation for participation

Participants who completed interviews were offered a pre-paid mobile phone card called a Hala Card in the amount of 100 QR ~ $30 USD, as compensation for their participation. Compensation was not offered until the end of the interview because during pilot testing, concern emerged that the offer of compensation might offend individuals of financially sound backgrounds or others who were not accustomed to participating in research studies. The RAs were also concerned that offering the Hala cards at the beginning might put them under undue pressure to give compensation to individuals who left for appointments before completing the interview and who might not come back.

### Data analysis

Based on the RA field notes, we calculated percentages of participation as well as descriptive statistics of the enrolled participants. The audio-recorded interviews were transcribed in the native language, and the transcription checked by a second reviewer. To protect the privacy of individuals, place names and any possible identifying information were changed. For Arabic, Urdu, and Hindi, the transcripts were independently translated into English, compared for similarity, and differences were reconciled by consulting with a third bilingual researcher. Iteratively, three team members (AK, HE, and MF) immersed themselves in the data by reading and open coding transcripts independently to develop preliminary codes. They reviewed and refined the codes and definitions during regular meetings. Emergent themes were incorporated into the coding scheme, and coding definitions were refined using general consensus of team members. Two analysts (AK and HE) independently coded and compared the coding of two transcripts for calibration, while a third (MF) resolved any disagreements. With calibration complete, the primary analyst (AK) coded all remaining transcripts and had weekly consultations with team members to clarify any concerns. The qualitative analysis software ATLAS.ti [30] was used for data management and analysis procedures.

To make the large qualitative dataset manageable, data transformation was employed whereby the coded qualitative themes were counted. Major codes were used to generate a summary for review, and then the search was refined for output by language and gender. After the initial analysis of the textual data from the field observations and transcripts, and numerical data from the demographic instrument and recruitment procedures, we integrated these diverse sources of data into a narrative format for the results.

## Results

### Participant recruitment

The research assistants approached 153 individuals; after eligibility exclusions and patient declinations, a total of 84 individuals (43 women and 41 men) were included as research participants (Tables 2 and 3). Individuals were excluded primarily for not meeting sampling criteria (Table 4). Thirty (20%) individuals declined to participate. More women (n = 19) than men (n = 11) declined participation, with the highest number of declinations in the Arabic language group (n = 20), and the highest number total occurring among women in the Arabic language group (Tables 2 and 4). At times, no specific reason for declining was provided; primary reasons included concern about the length of the interview and concern about the interview being recorded (Table 4). Overall, the participation rate among those who were approached and eligible was 74% (84/114).

**Table 2:** Distribution of individuals approached, declined, excluded, and enrolled by language and gender

	**Approached (153) total**			**Declined**			**Excluded**			**Enrolled**		
	**Male**	**Female**	**Subtotal**	**Male**	**Female**	**Subtotal**	**Male**	**Female**	**Subtotal**	**Male**	**Female**	**Subtotal**
Arabic	20	32	52	5	15	20	4	4	8	11	13	24
English	12	13	25	1	0	1	1	3	4	10	10	20
Hindi	27	13	40	2	2	4	15	1	16	10	10	20
Urdu	18	18	36	3	2	5	5	6	11	10	10	20
**Total**	**77**	**76**	**153**	**11**	**19**	**30**	**25**	**14**	**39**	**41**	**43**	**84**
	50%	50%		37%	63%	20%	64%	36%	25%	49%	51%	55%

**Table 3:** Demographics of interview participants

**Variable**	**Definition**	**Count**	**%**
**Gender**			
Male		41	49
Female		43	51
**Age (years)**			
18-24		2	2
25-34		18	21
35-44		14	17
45-54		28	33
55-64		14	17
65-74		8	10
75 and older		0	0
**Education**			
No Education	No school and/or no formal education	7	8
Some primary	Some primary, elementary, grade school or equivalent	3	4
Primary	Completion of primary, elementary, grade school or equivalent	8	10
Some middle school	Some middle school or the equivalent	9	11
Middle School	Completion of middle school or the equivalent	2	2
Some High School	Some high school or equivalent	1	1
High School	High school graduate or equivalent	17	20
Some College	Some college or equivalent	7	8
College	College graduate or equivalent	14	17
Post-College	More studies after completion of college or equivalent	16	19
**Home Region**			
Northern Europe		8	10
Northern America		1	1
Northern Africa		8	10
Eastern Europe		1	1
South-Eastern Asia		5	6
Southern Europe		1	1
Southern Africa		0	0
Southern Asia		41	49
Western Europe		1	1
Western Africa		1	1
Western Asia		15	18
Central Asia		1	1
Australia and New Zealand		1	1
Years Lived in Qatar*			
0-5		22	26
6-10		17	20
11-20		11	13
21-30		17	20
31-40		5	6
40 <		10	12
Unknown		2	2
Religion*			
Muslim		53	63
Hindu		6	7
Christian		22	26
Something else		1	1
Prefer not to say		2	2

*Percentages do not add to 100% due to rounding.

**Table 4:** Reasons potential participants were excluded from or declined participation by language

**Reasons for exclusions from the research by language (n = 39)**						
		**Not fluent in target language**	*Other	**Did not meet literacy criteria**	**Total**	**Percent by language group**
Arabic		2	4	2	8	21%
English		1	3	0	4	10%
Hindi		10	2	4	16	41%
Urdu		3	7	1	11	28%
Total		16	16	7	39	
Percent		41%	41%	18%		
**Reasons for declining to participate in the research by language (n = 30)**						
	**Did not want to participate in interview**	**Declined due to time constraint**	**Did not want the recording**	**Other	**Total**	**Percent by language group**
Arabic	6	4	5	5	20	67%
English	0	1	0	0	1	3%
Hindi	3	0	1	0	4	13%
Urdu	2	3	0	0	5	17%
Total	11	8	6	5	30	
Percent^	37%	27%	20%	17%		

*Includes the following reasons: individual has been in Qatar less than a year, started but did not complete the interview, is not a patient, did not have experience in Hamad, had already been interviewed, recording equipment failed, patient was oxygenated and could not be interviewed, and recording was inaudible.

**Answers occurring once included individual being part of Hamad staff, being too tired to participate, had to ask permission from husband, prevented from participation by daughter, and unable to answer questions.

^Percentages do not add to 100% due to rounding.

Based on our analysis of the qualitative data, we identified a series of themes and sub-themes about recruitment, informed consent, and compensation as presented below.

#### Hesitation to participate

During recruitment, some individuals expressed hesitation when approached for enrollment in the study. Some (n = 6) individuals premised their consent to participate only after the RAs provided them with a guarantee that they would not have to share their name or reveal their identity; this occurred mainly in the Hindi language group (n = 5). Three participants were concerned about having to answer personal questions and needed assurance that they could refuse to answer any question during the interview.

During the recruitment and informed consent process, some individuals hesitated upon learning that the interview was going to be recorded. Among all individuals approached, twelve people (9 women and 3 men) expressed concern about the recording. Six of them declined for this reason, and four others hesitated but ultimately chose to participate. Among all language groups, more people in the Hindi (n = 5) and Arabic (n = 5) groups expressed concern, and more people in the Arabic group (n = 5) refused to participate in the interviews due to recording procedures. Some individuals (n = 8) were reluctant due to the length of the interview and needed to return to their jobs. As individuals were recruited while waiting for their appointments, some people (n = 3) hesitated due to fear of losing their turn to see the doctor.

#### Discussion of participation with a family member

A few women preferred to discuss whether or not to participate with a family member. Husbands played an essential role in the decision-making process in five instances. One woman declined participation because she needed to ask her husband for permission. Upon learning that the interview would be recorded, two women sought and were granted permission to participate by their husbands. As for the other two women, they consulted with their husbands, though it was unclear if they did so (1) to get permission, (2) to consider the husband’s flexibility due to dependency on him for transportation, (3) for consideration of timing/scheduling or (4) for assistance with the interview. Mothers and daughters also played a role in the decision-making process. In one case, a mother approved of her adult daughter’s participation, whereas another mother declined to participate after her two daughters discouraged her.

### Informed consent for interviews

Some participants read the information/waiver of written informed consent sheet; others asked the RAs to read it to them or provide them with a verbal overview due to being illiterate, not having their reading glasses, or being short on time. Of the 78 individuals tracked, 60 (77%) chose to keep the information/waiver of written informed consent sheet, and there was no identifiable difference by language or gender.

### Interview environment

#### Exceptions to gender rules

Although interviews with female participants were mostly conducted in the women’s waiting rooms and interviews with male participants in the men’s waiting rooms, there were some instances of flexibility relative to gender rules. In five instances, the RAs interviewed male participants in vacant patient rooms usually with the door open, without objections. Additionally, some participants and their family members used the waiting room of the other gender. For example, a woman was interviewed in the men’s waiting area because she wanted her husband to be present during the interview. In another example, a woman had her husband join her for the interview in the women’s waiting room after permission from the head nurse.

#### Privacy in the waiting area

Despite the location of the busy outpatient waiting room, most participants completed the interview. Some participants, more often women (n = 10) than men (n = 3), were accompanied by various people to their doctor’s appointment including spouses, mothers, children, relatives, maids/helpers, and friends. As “incidental research participants”, their participation occurred unexpectedly in a supportive way of the individual enrolled. At times they re-phrased questions and offered comments during the interview. The RAs felt that generally the presence of others did not inhibit participant responses; in one instance, an interview generated the interest of a patient who was sitting nearby. A potential downside was noted by one RA who felt that participants shared more information when interviewed in a separate room.

### Member checking

At the conclusion of the interview, participants were asked if they were interested in receiving information about the results of the study, a process called member checking [31]. Of all participants initially approached and who provided contact information, most were willing to provide their contact information to receive study results, with proportionally more participants in the Arabic group (n = 22) and less participants in the Hindi group (n = 8) doing so. Some people provided their address after the interview was completed and the recorder was turned off. Most participants (n = 48) shared their address in Qatar, though some shared an email, phone number, or address in their home country.

### Compensation for participation

There were various responses to presenting the compensation after the interview, ranging from gratefulness, reluctance, and outright declinations (Table 5). Despite the interviews being interrupted for appointments, most participants returned to complete the interview without knowing about the compensation.

**Table 5:** Responses of participants to offer of compensation

**Expressed gratitude.**	***RA:*** *Ah, thank you so much and to express my gratitude for your time and effort, I want to give you a fifty riyal Hala Card.*
Participants often thanked the RAs for the Hala card, sometimes making a prayer for them.	
	***P:*** *No [laughs].*
	***RA:*** *It’s not a big thing really.*
	***P:*** *No, no… My love.*
	***RA:*** *Thank you so much for your time.*
	***P:*** *God bless you.*
	Jordanian woman
**Exhibited surprise.**	***P:*** *Wow!*
Some participants were pleasantly surprised to receive the card.	***RA:*** *As a token of appreciation.*
	***P:*** *(Laughing) Wow!*
	***RA:*** *That you… you gave your important suggestions, it was your time was valuable still you waited for us to finish the interview.*
	***P:*** *That’s great.*
	Pakistani woman
**Engaged in polite bartering.**	***RA:*** *…. to express my thankfulness I would like to give you fifty riyals Hala Card for the time that you had spent (****S:*** *[laughs]) and the valuable information that you gave me…*
Others politely declined the card, resulting in a back and forth cultural exchange about whether or not they should accept the compensation. Some RAs playfully pleaded with participants to accept the cards as a reward for their participation.	
	***P:*** *I don’t need it really*
	***RA:*** *…no it’s just as a gift, a gift [laughs]*
	***P:*** *alright, I won’t say no*
	Bahraini man
	***RA:*** *… And as an appreciation for giving us the time, we would like to give you a Hala Card of fifty riyals.*
	***P, RA:*** *(Laughing)*
	***P:*** *Thank you, you don’t have to do that.*
	***RA:*** *No, it’s okay.*
	***P:*** *No, you have to have it.*
	***RA:*** *(Smiling) No, no, no.*
	***P:*** *No, no, you have to have it*
	***RA:*** *I can’t have it. I have to give it to you.*
	***P:*** *Oh, that’s a shame.*
	British woman
**Commented that compensation was unnecessary.**	***RA:*** *…This is for you…*
Some participants felt the cards were unnecessary, as they didn’t use them or should be given to others who are more in need.	***P:*** *What’s that, what’s that?*
	***RA:*** *For taking part in the research.*
	***P:*** *Give it to somebody else if he needs it.*
	British man
	*It’s alright. No, because I don’t use these cards.*
	British man
	*Okay, is this charity?*
	Pakistani man
**Clarified motivation for participation.**	*If at least one person gets a better treatment because of my sayings, I am happy. I am happ(ier) than this reward.*
Some participants clarified their motivation for participating in the study was for altruistic purposes.	
	Indian woman
	*This is as my duty, means, it is not…aah…I mean this is not about money at all.*
	Egyptian woman
**Stated they were unfamiliar with card.**	***P:*** *What is a Hala Card?*
A few people were unfamiliar with the Hala Card and did not know how to use it. The RAs explained that the Hala Cards are to be used with their mobile phones.	***RA:*** *For the mobile.*
	***P:*** *Okay.*
	Egyptian woman
	***RA:*** *…this is a simple appreciation, a present for your time and your collaboration…*
	***P:*** *What am I supposed to do with this?*
	***RA:*** *Use it.*
	***P:*** *Where to use it?*
	***RA:*** *This is Hala Card; use it for your telephone.*
	Palestinian woman
**Expressed concern about negative consequences.**	*No, still if I take this card then there is no problem for me?*
Some participants were concerned that accepting the reward at the end of the interview would result in negative ramifications for them.	
	Bangladeshi man
	*We will not fall into trouble because of this no?*
	Nepali man

RA = Research assistant, P = Participant.

### Motivation to participate

Motivation to participate varied among individuals. Some participants expressed disapproval of the health system and wanted to share their complaints, while others felt that the interview provided them an opportunity to share their experiences and offer recommendations. One of the most important motives for participating in the study was altruism. Participants expressed hope that their participation would help improve the system. One woman said, *“…if at least one person gets…better treatment…I am happy…That’s why when you asked me for this…research, I wanted to spend my time.”* Another added, *“…I feel obliged…I will also get the benefit…Because…’til the baby…cr(ies), mother don’t give milk. So, ‘til we tell they will not know. There won’t be improvement.”*

### Vulnerability

Despite many individuals being forthcoming with information about their experiences, a sense of vulnerability from participating emerged as a recurring theme throughout the recruitment and informed consent process among a sizeable minority of participants.

#### Name sharing

Through the normal discourse in a conversational style approach to interviewing, conversations sometimes shifted to the participant’s name. Most participants (n = 71, 85%) shared their names with the RAs, with men (n = 36) and women (n = 35) equally sharing. However, a minority of participants (n = 13, 15%) did not share with some expressing concerns about revealing their identity; participants in the Hindi language group (n = 8) were proportionately the least likely to share their name.

#### Interview recording

Individuals had a range of responses upon learning that the interviews would be recorded. During recruitment, some individuals declined to participate outright, and others consented after assurance from the RAs that the recordings would be deleted and that participants were not required to reveal their identity. Concern about the recording was also raised during the interview phase. In addition to the nine women who expressed concern during recruitment and informed consent as noted earlier, an additional five women expressed concern during the actual interview. There were concerns about the recording in all language groups, with the most concern expressed by individuals in the Arabic (n = 8) and Hindi (n = 7) language groups.

Some participants requested the RAs to pause the recording at different times during the interview, as illustrated by a Qatari man: “*Before- (I) raise this, I want to tell you a word, don’t record it.”* An Indian woman paused the recorder every time she wanted to share a negative experience: “*No. I don’t want all this to be recorded. I believe you but please don’t record. You can write down if you want.”* A British woman used hand gestures to convey negative feelings and did not complete her sentences, as observed by the RA: “*she…(left her) sentences incomplete…and (used) gestures to avoid saying. . . words. The interviewer had to say these words so that these expressions also get recorded. . . She spoke more openly when (the) conversation was not being recorded.”* Some participants shared more about their experiences after the interview was finished and the recorders were turned off. For example, a Pakistani woman shared a negative experience, but would not allow the RA to turn the recorder back on, even though she had no objection to the RA writing about it in her presence.

#### Concern about negative consequences

A few people expressed concern about participation in the study resulting in negative consequences for them or their families. Among all 153 individuals approached, four women and three men expressed this concern, with the issue being raised mainly in the Hindi language group (n = 5), followed by the Urdu (n = 1) and English (n = 1) groups, and no observed references in the Arabic group. Concern about negative repercussions was expressed at multiple points during the interview process, specifically during recruitment, the interview, member checking, and compensation.

During the interviews, a few participants were cautious in voicing negative feedback about the healthcare system. A British woman apologized for giving generalized answers, as she was worried about where her answers might end up. A Pakistani woman wondered about her husband’s job being impacted. An Indian woman was cooperative, but was not willing to fully share when the recorder was on ‘*because they may consider it as a complaint and something will happen to us.’* She incidentally ran into the RA a few days after the interview, and inquired about sharing further information.

Concerns about negative consequences emerged even with inquiries about member checking as two participants refused to provide their contact information to receive the study results. A Pakistani man cited concern about his visa being cancelled, and a Bangladeshi man seemed conflicted and eventually, he decided not to share his address:

*“No, if I give my address it is not good no? …If something wrong happens to me then it is not correct to give it no?…I will not give. I have all addresses. I have (in) Bangladesh, (in) Qatar also…I am (a) company person (company worker…Now if…. I give you my address, then what will people say? Eating food of Qatar and not saying good to it. He is saying that India, Bangla (desh) is good…That’s why I don’t want to give.”* During the compensation phase, two participants were concerned that accepting the Hala Card would result in negative ramifications as expressed by a Nepali man, “*We will not fall into trouble because of this, no?”* Even though the majority of participants were willing to give their suggestions for improving healthcare, a minority of participants conveyed a sense of vulnerability.

## Discussion

There are few empirical studies on recruitment, informed consent, compensation, and other research procedures [3] in countries other than the United States and Western Europe, with none in particular to the Middle East and Arabian Gulf other than a single study with adolescent refugees [18]. To our knowledge, this is the first study in the Middle East and Arabian Gulf to provide empirical data on recruitment and informed consent procedures in a general adult population invited to participate in research. Our study also provides the only empirical data available about the responses of participants in the Middle East and Arabian Gulf to compensation and member checking procedures.

We noted some interesting trends relevant to recruitment. More individuals in the Arabic language group declined participation than in any other language group, and three times more women than men in this group declined. As the number of declinations by men were close to the other groups, it is difficult to discern if there is a cultural pattern. However, the declinations among women were greater among Arabic speaking women than all other groups. One possible explanation can be found in the results from women who volunteered a reason for declining, and several individuals felt compelled to discuss with a family member whether to participate. While there were nine Qataris enrolled in the study, overall we did not notice any patterns suggesting differences between them and other Arabic speaking individuals. Future research could provide further information about gender and cultural differences.

We believe our success in recruitment, informed consent procedures, and participant enrollment was possible because we used culturally adapted procedures. We utilized the expertise of the Qatari research team members to ensure implementation of recruitment and consent procedures that were culturally adapted to Qatar’s cultural, political, and social practices [1]. We also employed culturally competent and language concordant RAs familiar with the cultural dynamics of individuals living in Qatar.

With regard to gender, we employed all-female RAs primarily to avoid breaching cultural sensitivities about gender interactions. Utilizing male RAs to recruit female individuals is more likely to affront cultural sensitivities about gender interactions and thus have a negative impact on the research, compared to female RAs recruiting male individuals. Although we had to recruit in gender specific waiting areas, having the female RAs wear white research coats conveyed their official status and helped to temper cultural guidelines of gender separation. Our RAs were also cognizant of the fact that in many non-Western collectivist societies, individuals may be bound to others in a web of social relations which can impact decision-making processes [18]. Thus, the RAs allowed individuals, especially women, to seek input from family members before participation, and they allowed family members to contribute to the interviews as “incidental research participants” if acceptable to the enrolled participant. While the team considered whether incidental research participants should provide separate informed consent, the team felt this would be too burdensome and disruptive to the discussions as they naturally unfolded.

Approximately half of all recruited individuals had low literacy, but we overcame this by having the RAs work directly with participants in their own language. Generally, we did not encounter difficulties obtaining informed consent from low literacy individuals in Qatar, in contrast to previous findings [20]. Although most participants retained the information/waiver of written informed consent sheet, we are unsure if the others were just not interested, preferred not to retain any linkage to the study, could not read, or had other reasons. We obtained verbal consent instead of written informed consent because in many non-Western societies, signatures are usually reserved for formal transactions associated with major life events [18]. Requests for written consent could rouse suspicion or concern, and asking illiterate participants to sign documents they are unable to read or fully comprehend can be threatening [4] or imply lack of trust [23]. Signatures would leave a record of participation, so individuals who preferred anonymity would have been precluded from participating. Interestingly however, we found that enrolled participants did not hesitate to provide their verbal consent despite it being audio-recorded. If executed as intended, written and verbal consent are ethically equivalent; thus, written consent shouldn’t be necessary. The willingness to provide verbal consent while hesitating to provide written consent illustrates the cultural aversion to signing documents.

To summarize our observations on informed consent procedures, we found culturally sensitive approaches to include: being flexible relative to family involvement in the consent process, explaining the study in the simplest terms possible to avoid overwhelming participants with too much information while still allowing them to make an informed choice, having forms in and interviewers who speak participants’ languages, being prepared for researchers in the field to read informed consent materials, avoiding written requirement for signature or substituting verbal consent when appropriate, and using waiver of informed consent when appropriate.

Our study is the first to offer empirical data about the responses of participants in Qatar to compensation and member checking procedures. Most returned to complete the interview despite having no knowledge about the compensation, although there were varying degrees of interest in accepting the compensation. The data demonstrate that there were some participants who were taken aback and adamantly declined compensation. Unanswered questions remain as to the optimal timing and location of offering compensation. As for the member checking invitation, most participants were willing to provide contact information to receive study results. There was a difference between groups, with participants in the Arabic group most often sharing their contact information and the Hindi group least often doing so. This may reflect a greater sense of vulnerability among participants in the Hindi group.

Cultural adaptations also meant compromising privacy during interviews to respect cultural norms around gender interaction and to alleviate participants’ fears of losing their place in queue for their appointment. Although Western ethical standards may suggest privacy to be critical, we found that conducting interviews in a public space generally did not inhibit the responses of the majority of our participants. Despite the gender norms of the country, we found that in the five instances of female RAs interviewing male participants in a room, individual preferences ultimately determined whether a participant was comfortable being interviewed in private. Gender norms may not have applied as rigidly since the RAs were viewed as part of the healthcare staff and because it is more acceptable for men and women to interact in healthcare settings when there is no reasonable alternative.

Although many participants openly discussed their opinion of the healthcare system, a few expressed trepidation that their participation might result in negative consequences for them or their families. We suspect that vulnerability fears may be due to a number of reasons. First, many of the participants were expatriate workers from countries where human rights cannot be assumed, and this lens could inevitably carry into Qatar. Second, there is relatively less public knowledge about research participation in the Arabian Gulf and in many of the developing countries that the expatriates call home, so lack of familiarity with research could cause mistrust. Third, it is possible that some individuals may have had negative experiences in society, e.g. in the workplace, and through this lens interpreted that their research experiences might be similar. Fourth, particularly in the case of a working spouse, some may not want to contradict or compromise preferences or policies of a spouse’s employer. The RAs quickly learned the importance of confidentiality of personal information, and adapted by emphasizing that private information would not be collected and that information given could not be linked to the participants. Regardless of the low probability of negative implications for such individuals, it is important for researchers working in the Arabian Gulf, especially given so many expatriates, to be aware of how people may perceive the consequences of participating in research. We recommend that research procedures be designed to address the underlying concerns that participants, even though a minority, may have about vulnerability throughout all stages of the process. Overall, we did find that many participants felt empowered to provide information to the researchers and appreciated the opportunity to discuss their experiences and offer recommendations to improve the Qatari health system.

Investigators conducting international health research may face multiple challenges during the research process, particularly if Western ethical standards are used without consideration of local traditions and social contexts. Table 6 provides recommendations for researchers conducting research in the Arabian Gulf Region.

**Table 6:** Considerations for recruitment and consent in the Arabian Gulf

***Issue***	***Context***	***Recommendation***
Hesitation to participate during recruitment	Individuals may have multiple concerns about participation.	-Assure individuals that sharing one’s name is not necessary, and avoid collecting such information unless critical to the study.
		-Advise subjects the researchers will not disclose any personal information the participant is not comfortable disclosing.
		-Refrain from audio and video recordings if not essential, and consider offer of hand written field notes only.
		-Reassure individuals that participating will not impact their care.
Informed Consent	Some non-Western communities reserve signing of documents for formal events.	-Be flexible relative to family involvement.
		-Explain the consent process to participants but don’t overwhelm participants with too much information.
		-Allow for verbal consent or waiver of written informed consent.
		-Consider language and literacy barriers; be prepared with bilingual documents and interviewers.
Circumstances of where and when to interview	Cultural rules may dictate gender separation.	-Consider corner of waiting area with option for a private room as safe place where interviews can occur between men and women in a publicly visible place.
		-Allow participants to stay in the waiting room to alleviate their fears of losing their turn.
		-Allow participants to finish interviews after their appointment if needed.
		-Trust local staff to make the decision to choose location.
Involvement of family members as “incidental research participants”	In non-Western communities, decision-making processes tend to be more collective than individualistic.	-Allow individuals to seek input from family members, and this could range from permission to participate, i.e., participant is subordinate to the person they discuss it with, otherwise it may be a negation of agreement or assent of a valued family member’s input.
		-Consider flexibility regarding boundaries around participation.
Member checking	Individuals may not be familiar with research studies. Some may be unable to provide their contact information.	-Invite participants to learn more about the results of the study.
		-Provide assurances of confidentiality.
Compensation	Offer of compensation could be offensive or unfamiliar.	-Consider discussion of compensation after interview is completed.
Vulnerability	Individuals may have vulnerability fears due to their sociocultural and residency status.	-Reassure participants that confidentiality of participation will be protected.
		-Emphasize safety of disclosing information.

There have been many efforts to establish national research ethics guidelines in the Middle East and Arabian Gulf, with most of the Arabian Gulf countries making more advanced steps than other countries in the Middle East [32]. Researchers are expected to balance universal ethical standards with local standards [8,32]; therefore, ethical research principles should be upheld in as much as they are appropriate to the local context [2,18]. We agree with Fadare and Porteri’s position, who state, “While international research ethics guidelines have an important role in setting basic and acceptable standards, there remains a window for local adaptation, especially in resource-poor and culturally diverse countries [2].”

All studies have possible limitations. As the study was conducted in one health care setting in Doha, the degree that recommendations from this study will hold in other settings needs further exploration. Our study employed all-female RAs; utilizing male RAs may have had different implications for the recruitment and informed consent process. Interviewing participants in a busy outpatient waiting room rendered data collection feasible, though this choice may have limited the information participants provided, such as criticisms or private information. Future research could compare outcomes such as participation rates and data quality when data collection occurs in public vs. private settings to clarify the advantages each setting might offer. While the RAs endeavored to observe carefully, there may have been other meaningful events that they did not notice or document. Finally, qualitative text from interviews was volunteered by the participants in response to open-ended questions, so the reported numbers represent minimum estimates when qualitative responses were quantified. Caution should be exercised in generalizing the prevalence of the various positions stated since participants were not randomly sampled.

## Conclusion

This empirical study from Qatar provides the first comprehensive consideration of recruitment, informed consent, compensation, and other research procedures in Middle East and Arabian Gulf Region. Based on these findings, we conclude that Western approaches to recruitment, consent, and compensation are compatible in Qatar but with the caveat that researchers should consider potential cultural influences. Specific issues meriting consideration include the data collection environment, gender relations, preferred informed consent procedures, sensitivity about compensation, possible involvement of family members as incidental research participants, and privacy concerns due to a perceived sense of vulnerability. With attention to these cultural influences, our investigation achieved a very good participation rate. Given the limited literature about research participation in the Middle East and Arabian Gulf Region, additional studies on the perspectives of researchers and research participants would be helpful in exploring the challenges of conducting research in non-Western contexts, abiding by Western IRB standards, and ethically incorporating cultural adaptations. We conclude that researchers embarking on health research in the Middle East and Arabian Gulf Region should consult closely with colleagues on the ground to assess and account for local cultural sensitivities.

## Competing interests

The authors declare that they have no competing interests.

## Authors’ contributions

AK conducted the qualitative analysis and drafted the manuscript. AKh assisted in drafting the manuscript and along with ME and HA, participated in the design and coordination of the study. HA and HAs participated in data collection. MH participated in the design of the study. MT participated in coordination of the study. HE assisted with data analysis. AA assisted with design and recruitment. MF conceived of the study, participated in its design and coordination, assisted with analysis, and helped to draft the manuscript. All authors read, revised, and approved the final manuscript.

## Pre-publication history

The pre-publication history for this paper can be accessed here:

http://www.biomedcentral.com/1472-6939/15/9/prepub
